# Acute and Chronic Administrations of *Rheum palmatum* Reduced the Bioavailability of Phenytoin in Rats: A New Herb-Drug Interaction

**DOI:** 10.1155/2012/701205

**Published:** 2012-07-08

**Authors:** Ying-Chang Chi, Shin-Hun Juang, Wai Keung Chui, Yu-Chi Hou, Pei-Dawn Lee Chao

**Affiliations:** ^1^Institute of Pharmaceutical Chemistry, China Medical University, Taichung 40402, Taiwan; ^2^Department of Pharmacy, Faculty of Science, National University of Singapore, 18 Science Drive 4, Singapore 117543; ^3^School of Pharmacy, China Medical University, Taichung 40402, Taiwan; ^4^Department of Medical Research, China Medical University Hospital, Taichung 40402, Taiwan

## Abstract

The rhizome of *Rheum palmatum* (RP) is a commonly used herb in clinical Chinese medicine. Phenytoin (PHT) is an antiepileptic with narrow therapeutic window. This study investigated the acute and chronic effects of RP on the pharmacokinetics of PHT in rat. Rats were orally administered with PHT (200 mg/kg) with and without RP decoction (single dose and seven doses of 2 g/kg) in a crossover design. The serum concentrations of PHT, PHT glucuronide (PHT-G), 4-hydroxyphenytoin (HPPH), and HPPH glucuronide (HPPH-G) were determined by HPLC method. Cell line models were used to identify the underlying mechanisms. The results showed that coadministration of single dose or multiple doses of RP significantly decreased the *C*_max_ and AUC_0-t_ as well as the *K*_10_ of PHT, PHT-G, HPPH, and HPPH-G. Cell line studies revealed that RP significantly induced the P-gp-mediated efflux of PHT and inhibited the MRP-2-medicated transport of PHT and HPPH. In conclusion, acute and chronic coadministrations of RP markedly decreased the oral bioavailability of PHT via activation of P-gp, although the MRP-2-mediated excretion of PHT was inhibited. It is recommended that caution should be exercised during concurrent use of RP and PHT.

## 1. Introduction

The rhizome of *Rheum palmatum* (RP, Dahuang) is one of the important herbs widely used in China, Russia, and Arabia [1]. The major constituents of RP include a variety of anthraquinones such as aloe-emodin, rhein, emodin, chrysophanol and physcion [2, 3], which have been reported to show various beneficial effects, including neuroprotective, antioxidant, anti-inflammatory, and anticancer activities [4–8]. Recent pharmacokinetic studies of RP have revealed that the anthraquinones were all predominantly present as glucuronides and sulfates in the blood [3, 8] and they are also putative substrates of multidrug resistance proteins (MRPs), namely, the anion transporters.

Phenytoin (PHT), a widely used antiepileptic with narrow therapeutic window, follows nonlinear pharmacokinetics, and thus therapeutic drug monitoring is usually recommended during its use [9]. The adverse reactions of PHT include drowsiness, dysarthria, tremor, and cognitive difficulties [10, 11]. PHT has been reported as a substrate of P-glycoprotein (P-gp) and MRP 2, whose expressions determined the PHT level in brain [12, 13]. PHT is metabolized to its main metabolite 4-hydroxyphenytoin (HPPH) by cytochrome P450 (CYP) 2C9 and to a minor extent by CYP 2C19 [14, 15]. Both PHT and HPPH are metabolized by glucuronidation to form PHT glucuronide (PHT-G) and HPPH glucuronide (HPPH-G), respectively [16, 17].

Based on our understanding on the metabolic fates and pharmacokinetics of PHT and anthraquinones in RP, we hypothesized that the metabolites of anthraquinones might compete with PHT, HPPH, PHT-G, or HPPH-G for anion transporters such as MRP 2. Patients suffering from epilepsy are generally dependent on life-long antiepileptic treatment. On other hand, using RP for constipation is an excellent home remedy in oriental countries. Therefore, it is probable that epileptic patients combined the use of PHT and RP. As such coadministration of RP and PHT may give rise to adverse effects, therefore, this study was set up to investigate the acute and chronic effects of coadministration of RP on the pharmacokinetics of PHT in rats. In addition, cell line models would be used to explore the underlying mechanism of this herb-drug interaction.

## 2. Materials and Methods

### 2.1. Chemicals and Reagents

PHT (purity 99%), HPPH (purity 98%), aloe-emodin (purity 95%), rhein (purity 95%), emodin (purity 95%), chrysophanol (purity 98%), physcion (purity 98%), verapamil (purity 99%), indomethacin (purity 98%), propylparaben (purity 99%), 2-methylanthraquinone (purity 95%), rhodamine 123 (purity 99%), and 3-(4,5-dimethylthiazol-2-yl)-2,5-diphenyl tetrazolium bromide (MTT) were purchased from Sigma Chemical Co. (St. Louis, MO, USA). L(+)-Ascorbic acid was obtained from Riedel-de Haën Laborchemikalien GmbH & Co. KG (Seelze, Germany). Fetal bovine serum was supplied by Biological Industries Ltd., Kibbutz Beit Haemek, Israel). L-Glutamine, penicillin, streptomycin, nonessential amino acid, trypsin-EDTA, and Hank's balanced salt solution (HBSS) were purchased from Invitrogen Inc. (Carlsbad, CA, USA). Total protein assay kit was purchased from Bio-Rad Inc. (Mississauga, ON, Canada). Other reagents were HPLC grade or analytical grade. Milli-Q plus water (Millipore, Bedford, MA, USA) was used throughout this study.

### 2.2. Preparation and Characterization of RP Decoction

The crude drug of RP was purchased from a Chinese drugstore in Taichung, Taiwan. The origin was identified by Dr. Yu-Chi Ho via microscopic examination. A voucher specimen (CMU-P-1905-9) was deposited in the College of Pharmacy, China Medical University. Water (5 L) was added to 250 g of the crude drug. After maceration for 1 h, the mixture was heated to boiling and gentle heating was continued for about 2 h until the volume was reduced to less than 2.5 L. The mixture was filtered while hot and the filtrate was concentrated further by gentle boiling until the volume was reduced to below 500 mL, after which sufficient water was added to make 500 mL (0.5 g/mL of RP). The resultant concentrate was divided into aliquots of 40 mL and stored at −20°C for later use.

The concentrations of aloe-emodin, rhein, emodin, chrysophanol, and physcion in RP decoction and its hydrolysate were determined by an HPLC method. For acid hydrolysis, a portion of the decoction (1.0 mL) was added 1.2 N HCl (1 mL), 25 mg of ascorbic acid and incubated at 80°C for 30 min. This method was determined by a previous preliminary study. The mixture was then added with 4.0 mL of methanol. After vortexing and centrifugation, the supernatant (100 *μ*L) was mixed with 100 *μ*L of internal standard in the form of 2-methylanthraquinone solution (50 *μ*g/mL in methanol) and 20 *μ*L of this solution was subjected to HPLC analysis. A gradient elution was carried out using a mobile phase consisting of 0.1% phosphoric acid (A) and acetonitrile (B) that were mixed in the following program: A/B = 50/50 (0–10 min), 15/85 (15–22 min), 50/50 (27–30 min). The detection wavelength was set at 280 nm and the flow rate was maintained at 1.0 mL/min. The concentration of anthraquinone glycoside was estimated from the difference of aglycone concentrations between RP decoction and its acid hydrolysate.

### 2.3. Animals and Drug Administration

Male Sprague-Dawley rats were supplied by National Laboratory Animal Center (Taipei, Taiwan) and kept in the animal center of the China Medical University (Taichung, Taiwan). The animal protocol was approved by the Institutional Animal Care and Use Committee of the China Medical University. The animal study was conducted with adherence to “The Guidebook for the Care and Use of Laboratory Animals” published by the Chinese Society of Animal Science, Taiwan. The rats (300–400 g) were fasted for 12 h before drug administration. PHT was dissolved in dil. NaOH to afford a solution of 20.0 mg/mL [18]. PHT was given via gastric gavage to six rats at 200.0 mg/kg with and without a concomitant oral dose of RP decoction (single dose and seven doses of 2 g/kg) in a crossover design. The RP decoction was administered right before PHT.

### 2.4. Blood Collection

Blood samples (0.5 mL) were withdrawn via cardiac puncture at time points of 0, 15, 30, 60, 120, 240, 480, and 720 min after oral administration of PHT. The blood samples were collected in microtubes and centrifuged at 10,000 g for 15 min to obtain the serum, which was stored at −70°C before analysis.

### 2.5. Determination of PHT and Its Metabolites in Serum

For the determination of free forms of PHT and HPPH, 100 *μ*L of serum sample was mixed with 50 *μ*L of pH 5 acetate buffer and 50 *μ*L of ascorbic acid (100 mg/mL). The mixture was extracted with 200 *μ*L of ethyl acetate (containing propylparaben as internal standard, 5 *μ*g/mL). The ethyl acetate layer was evaporated under N_2_ to dryness and reconstituted with an appropriate volume of acetonitrile and then subjected to HPLC analysis.

For the assay of PHT-G and HPPH-G in serum, indirect determination was carried out through hydrolysis with *β*-glucuronidase in pH 5 acetate buffer. Serum sample (100 *μ*L) was mixed with 50 *μ*L of *β*-glucuronidase (1000 units/mL) and 50 *μ*L of ascorbic acid (100 mg/mL) and incubated at 37°C for 60 min under anaerobic condition. After hydrolysis, the analytical procedures followed that for the assay of the free forms as described above.

### 2.6. Cell Lines and Cell Culture

LS 180, the human colon adenocarcinoma cell line, was obtained from the Food Industry Research and Development Institute (Hsinchu, Taiwan). The nontransfected and MRP-2-overexpressing MDCK II cell lines were kindly provided by Professor Piet Borst (Dutch Cancer Institute, Amsterdam, The Netherlands). The cell lines were cultured in DMEM medium supplemented with 10% fetal bovine serum, 0.1 mM nonessential amino acid (LS 180 cell line only), 100 units/mL of penicillin, 100 *μ*g/mL of streptomycin, and 292 *μ*g/mL of glutamine. Cells were grown at 37°C in a humidified incubator containing 5% CO_2_. The medium was changed every other day, and the cells were subcultured until 80–90% confluency was reached.

### 2.7. Cytotoxicity Assay

Verapamil hydrochloride was dissolved in water. PHT and HPPH were dissolved in dil. NaOH (pH 9.0). Indomethacin (Indo) was dissolved in MeOH and the final concentration of MeOH in medium was below 0.1% (v/v). LS 180, MDCK II, and MDCK II-MRP 2 cells (1 × 10^5^ cells/well) were seeded into 96-well plates. After overnight incubation, the test agents were added into the wells and incubated for 72 h and then 15 *μ*L of MTT (5 mg/mL) was added into each well and incubated for additional 4 h. During this period, MTT was reduced to formazan crystal by live cells. An acidic SDS (10%) solution was added to solubilize the purple crystal formed at the end of incubation and the optical density was measured at 570 nm by a microplate reader (Nunc, Denmark).

### 2.8. Transport Study of PHT in LS 180

LS 180 cells (passage 50 to 60) were seeded on 12-well plates at a density of 5 × 10^5^ cells/well. Before the experiment, the medium was removed and the cells were quickly rinsed with ice-cold HBSS transport buffer consisting of HEPES (10 mM, pH 7.4.). PHT (10 *μ*M) was coincubated with and without RP (2.0, 1.0 and 0.5 mg/mL) and verapamil (100 *μ*M) with LS 180 for 90 min. After incubation, the cells were rapidly washed twice with ice-cold HBSS buffer and lysed with 200 *μ*L of 0.1% Triton X-100 for 30 min.

Total protein content in the lysate (5 *μ*L) was determined by Bradford method [19], and bovine serum albumin was used as the standard. The protein contents in each well were used for data correction.

### 2.9. Preparation and Characterization of Serum Metabolites of RP (RPMs)

In order to mimic the molecules interacting with MRP 2 in the kidney, RPMs was prepared using rats. After overnight fasting, rats were given RP decoction at 2 g/kg. Blood was collected at 30 min after dosing. The serum was added with 3-fold methanol. After vortex and centrifugation at 10,000 g for 15 min, the supernatant was dried in a rotatory evaporator under vacuum. To the residue, an appropriate volume of water was added to afford a solution with a 10-fold serum concentration, which was divided into aliquots and stored at −80°C for later use. The procedures for the characterization of RPMs followed that of a previous study [2, 3]. Briefly, 100 *μ*L of serum sample were mixed with 50 *μ*L of sulfatase solution (containing 1000 units/mL of sulfatase and 35,600 units/mL of *β*-glucuronidase) and 50 *μ*L of ascorbic acid (100 mg/mL) and incubated at 37°C for 10 min under anaerobic condition. After hydrolysis, the serum was acidified with 50 *μ*L of 0.1 N HCl and extracted with 250 *μ*L of ethyl acetate (containing 2-methylanthraquinone as internal standard, 1 *μ*g/mL). The ethyl acetate layer was evaporated under N_2_ to dryness and reconstituted with an appropriate volume of methanol prior to HPLC analysis. In addition, the serum of rats given water only was collected to prepare the blank control. The blank serum was processed as described above and diluted to various folds of serum concentration for the comparison with correspondent concentration of RPMs.

### 2.10. Transport Study of PHT and HPPH in MDCK II and MDCK II-MRP 2

MDCK II and MDCK II-MRP 2 cells within 10 passages were seeded on 12-well plates at a density of 3 × 10^5^ cells/well. Before experiment, the medium was removed and the cells were quickly rinsed with ice-cold HBSS transport buffer. To determine whether PHT and HPPH are substrates of MRP 2, PHT (10 *μ*M), HPPH (10 *μ*M) and Indo (50, 100 and 200 *μ*M, as a positive control of MRP 2 inhibitor) were incubated with MDCK II and MDCK II-MRP 2 for 60 min.

In another study, RPMs and Indo (100 *μ*M) in HBSS were preincubated with MDCK II-MRP 2 for 30 min. The supernatants were removed and cells were washed three times with ice-cold PBS. Then, PHT (10 *μ*M) and HPPH (10 *μ*M) were coincubated with and without RPMs (1.0-, 0.5-, and 0.25-fold of serum concentrations) and Indo (200 *μ*M) with MDCK II-MRP 2 for another 30 min. After incubation, the cells were rapidly washed twice with ice-cold HBSS buffer and lysed with 200 *μ*L of 0.1% Triton X-100 for 30 min.

Total protein content in the lysate (5 *μ*L) was determined by Bradford method [19], and bovine serum albumin was used as the standard. The protein contents in each well were used for data correction.

### 2.11. Determination of PHT and HPPH Concentrations in Cell Lysate

For the assay of PHT and HPPH, 200 *μ*L of cell lysate was extracted with 200 *μ*L of ethyl acetate (containing propylparaben as internal standard, 5 *μ*g/mL). The ethyl acetate layer was evaporated under N_2_ to dryness and reconstituted with an appropriate volume of acetonitrile, then subjected to HPLC analysis.

### 2.12. Assay and Method Validation of PHT and HPPH in Serum and Cell Lysate

An HPLC method using a mixture of methanol and 0.05% phosphoric acid (48 : 52) as mobile phase was developed and validated for the assay of PHT and HPPH in serum and cell lysate. The detection wavelength was set at 214 nm and the flow rate was 1.0 mL/min. The calibration ranges of PHT and HPPH were 0.2–50.0 *μ*g/mL for serum and cell lysate. The precision and accuracy of the analytical method was evaluated by intraday and interday analysis of triplicate standards within one day and over a period of three days. Recoveries from serum were calculated based on the detected concentrations in serum compared with those in water. The mixture was extracted with 200 *μ*L of ethyl acetate containing 5 *μ*g/mL of propylparaben as internal standard. The ethyl acetate layer was evaporated under N_2_ to dryness and reconstituted with an appropriate volume of acetonitrile and then subjected to HPLC analysis. LOQ (Limit of Quantitation) represents the lowest concentration of analyte that can be determined with acceptable precision and accuracy with coefficients of variation and relative errors below 15% and 20%, respectively. LOD (Limit of Detection) represents the lowest concentration of analyte that can be detected with S/N > 3.

### 2.13. Data Analysis

The areas under the serum concentration-time curves (AUC_0-t_) of PHT, PHT-G, HPPH, and HPPH-G were calculated using noncompartment model (version 1.1, SCI software, Statistical Consulting, Inc., Apex, NC, USA). The peak serum concentrations (*C*_max⁡_) were from experimental data. One-way ANOVA with Scheffe's test was used for statistical comparison taking *P* < 0.05 as significant.

## 3. Results

### 3.1. Characterization of RP Decoction

**Figure 1:**
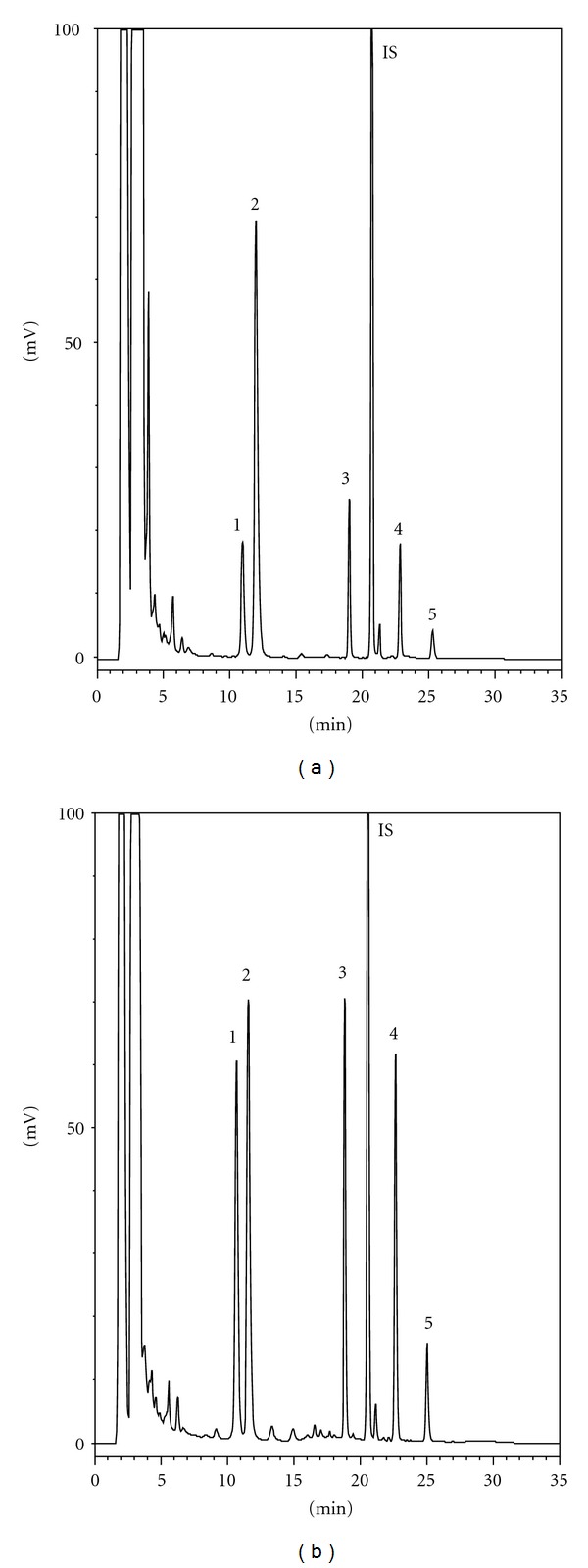
HPLC chromatograms of RP decoction before (a) and after (b) acid hydrolysis. 1: aloe-emodin, 2: rhein, 3: emodin, 4: chrysophanol, 5: physcion, IS: 2-methylanthraquinone.

Figure 1 shows the chromatograms of the RP decoction before and after acid hydrolysis. Quantitation results showed that the concentrations of aloe-emodin, rhein, emodin, chrysophanol, and physcion were 0.9, 2.0, 0.5, 0.4, and 0.1 nmol/mL in the decoction and 2.3, 3.8, 2.0, 1.8, and 0.7 nmol/mL in the acid hydrolysate of decoction, respectively. Accordingly, a dose of 2 g/4 mL/kg RP was found to contain 9.2, 15.2, 8.0, 7.2, and 2.8 nmol/kg of aloe emodin, rhein, emodin, and chrysophanol with the relevant glycosides, respectively.

### 3.2. Assay of PHT and HPPH in Serum and Method Validation

In serum assay, the calibration curves of PHT and HPPH showed good linearity in the concentration range of 0.2–50 *μ*g/mL. The precision evaluation revealed that all coefficients of variation were below 15% and the accuracy analysis showed that the relative errors to the true concentrations were below 10%. The recoveries of PHT and HPPH from serum were 95.1–100.8% and 94.1–99.0%, respectively. The LLOQ of PHT and HPPH was 0.4 *μ*g/mL and the LOD was 0.01 and 0.02 *μ*g/mL, respectively.

### 3.3. Effect of RP on PHT Pharmacokinetics in Rats

**Figure 2:**
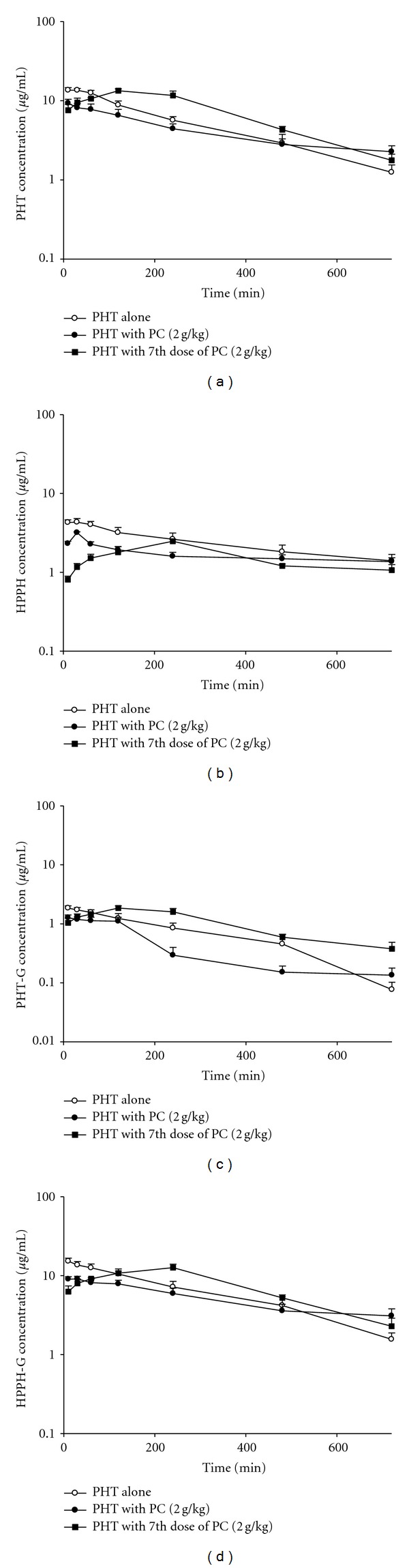
Mean (±S.E.) serum concentration-time profiles of PHT (a), HPPH (b), PHT-G (c), and HPPH-G (d) after oral administration of PHT alone (200 mg/kg) (°), coadministration with single dose (●), and 7th dose of 2 g/kg (■) of RP decoction in six rats.

Figure 2 depicts the mean serum concentration-time profiles of PHT, PHT-G, HPPH, and HPPH-G after oral administration of PHT alone and oral coadministration with single dose and pretreatment with seven doses of 2 g/kg of RP. The pharmacokinetic parameters of PHT, PHT-G, HPPH, and HPPH-G are listed in Table 1. Coadministration of RP with single dose of 2 g/kg significantly decreased the *C*_max⁡_ of PHT, PHT-G, HPPH and HPPH-G by 51.0, 51.7, 43.9, and 42.5% and reduced the AUC_0-720_ by 36.9, 51.7, 30.3, and 22.8%, respectively. Pretreatment with seven doses of RP significantly decreased the *C*_max⁡_ of PHT, PHT-G, HPPH, and HPPH-G by 53.1%, 65.1%, 46.3%, and 44.3% and reduced their AUC_0-720_ by 51.9%, 64.8%, 37.5% and 30.6%, respectively. In addition, the *K*_10_ of PHT, PHT-G HPPH, and HPPH-G were significantly decreased upon acute and chronic coadministrations of RP.

### 3.4. Cytotoxicity Assay

More than 90% of cells were viable at the concentrations of PHT, HPPH and RP up to 10 *μ*M, 10 *μ*M, and 2 mg/mL in LS 180, respectively. In addition, PHT, HPPH, and RPM at 10 *μ*M, 10 *μ*M, and 1.0-fold serum concentration, respectively, did not possess any noticeable cytotoxicity against MDCK II and MDCK II-MRP 2.

### 3.5. Transport Studies of PHT and HPPH

**Figure 3:**
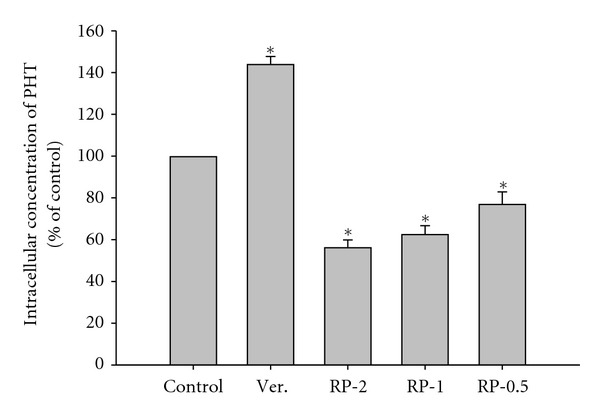
Effects of RP (2.0, 1.0 and 0.5 mg/mL) and verapamil (Ver, 100 *μ*M as a positive control of P-gp inhibitor) on the accumulation of PHT in LS 180 cells. Data expressed as mean ± S.D. of four determinations. *P < 0.05.

The effects of RP and verapamil on the intracellular accumulation of PHT in LS 180 cells are shown in Figure 3. RP at 2.0, 1.0 and 0.5 mg/mL significantly decreased the intracellular accumulation of PHT by 43.6, 37.3, and 22.9%, indicating RP concentration-dependently activated the efflux function of P-gp.

**Figure 4:**
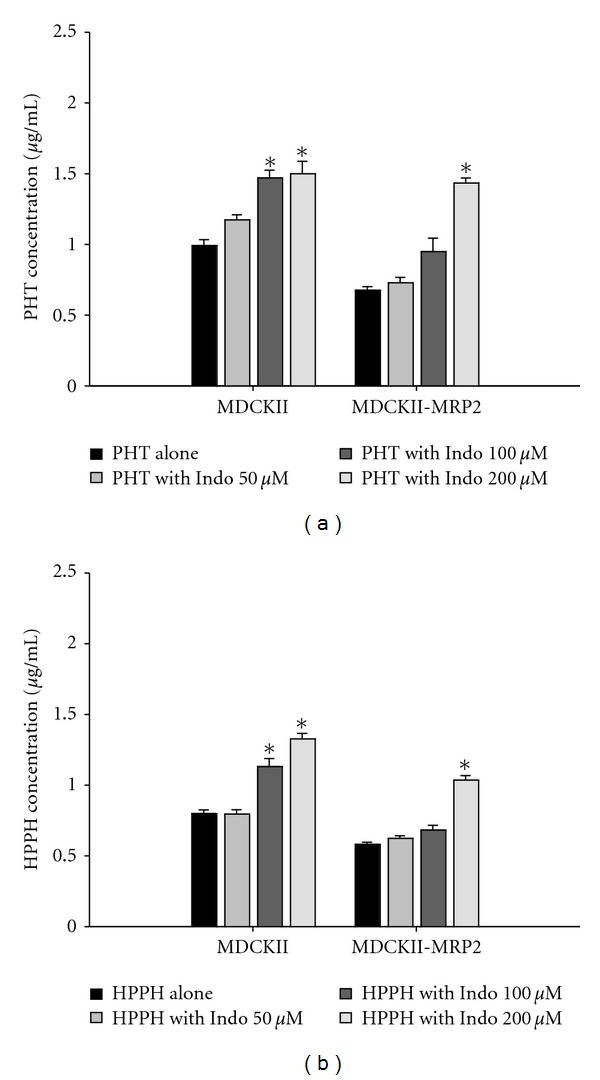
Effects of indomethacin (Indo) on the intracellular accumulation of PHT ((a), 10 *μ*M) and HPPH ((b), 10 *μ*M) in MDCKII and MDCKII-MRP 2 cells. Data expressed as mean ± S.D. of four determinations. *P < 0.05.

Figure 4 shows the differences of intracellular accumulation of PHT and HPPH in presence and absence of Indo in MDCK II and MDCKII-MRP 2. The results showed that the accumulations of PHT and HPPH in MDCKII were higher than those in MDCK II-MRP 2. In addition, the intracellular accumulations of PHT and HPPH were significantly increased by Indo in both cell lines.

In order to mimic the molecules interacting with MRP 2 in kidney, the RPMs of rats were prepared and characterized. HPLC analysis of RPMs showed that it contained 2.3, 13.0, 4.1, and 2.0 *μ*M of glucuronides/sulfates of aloe-emodin, rhein, emodin, chrysophanol, respectively, and 6.4 *μ*M of rhein free form in the serum.

**Figure 5:**
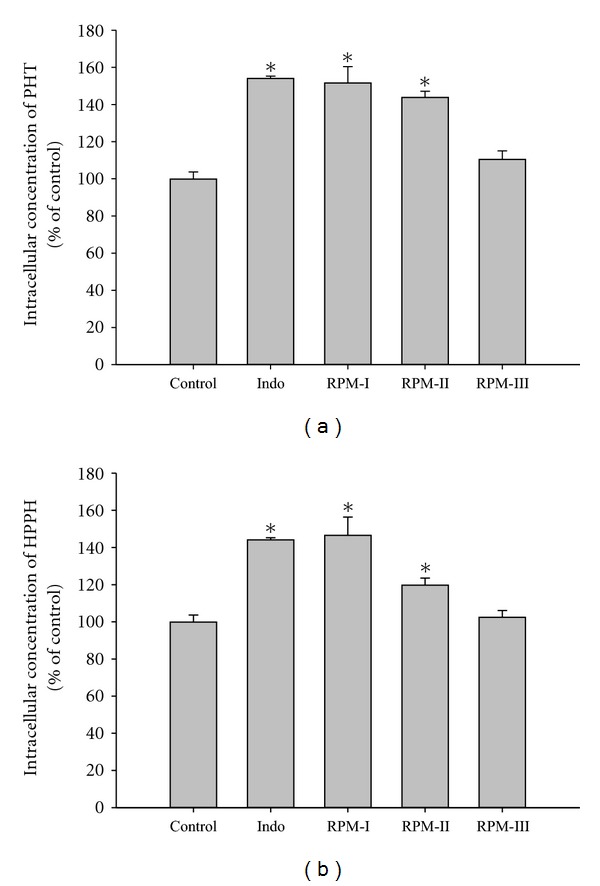
Effects of RP metabolites (RPMs) and indomethacin (Indo, 200 *μ*M as a positive control of MRP 2 inhibitor) on the accumulation of PHT (a) and HPPH (b) in MDCKII-MRP2 cells. Data expressed as mean ± S.D. of five determinations. *P < 0.05. RPMs-I, II, III: 1.0-, 0.5-, and 0.25-fold serum concentration, respectively (1.0-fold: containing 2.3 *μ*M of aloe emodin G/S, 6.4 *μ*M of rhein, 13.0 *μ*M of rhein G/S, 4.1 *μ*M of emodin G/S, and 2.0 *μ*M of chrysophanol G/S).

The effects of RPMs and Indo on the intracellular accumulation of PHT and HPPH in MDCK II-MRP 2 are shown in Figure 5. RPMs at 1-fold serum concentrations significantly increased the intracellular accumulation of PHT and HPPH by 51.7% and 46.7%. As a positive control, Indo at 200 *μ*M significantly increased the intracellular accumulation of PHT and HPPH by 54.2% and 44.1%, respectively.

## 4. Discussions

Owing to the general abundance of polyphenol glycosides in plants, characterization of the RP decoction used in this study was carried out to measure the concentrations of aloe-emodin, rhein, emodin, chrysophanol, and physicon before and after acid hydrolysis. Quantitation results showed that upon acid hydrolysis the concentrations of aloe-emodin, rhein, emodin, chrysophanol, and physicon increased by 416%, 85%, 345%, 402% and 367%, respectively, implying that the RP decoction contained aloe-emodin, emodin, chrysophanol, and physicon mainly in their glycoside form, whereas rhein was an exception existing more in the free form.

The assay methods of PHT and HPPH in serum and cell lysate were similar to a previously reported study but with some modifications made and they were validated in this study [17, 20]. The determination of PHT-G and HPPH-G was performed indirectly through hydrolysis with *β*-glucuronidase [21]. Coadministration of single dose and pretreatment with the seven doses of RP all significantly decreased the AUC and *C*_max⁡_ of PHT, PHT-G, HPPH, and HPPH-G, suggesting that RP decreased the oral bioavailability of PHT. The serum profiles revealed that the early exposure of PHT was markedly decreased, inferring that the absorption of PHT was hampered. This fact suggested that acute and chronic coadministrations of RP would result in diminished efficacy of PHT.

Based on previous studies which claimed that PHT was a substrate of P-gp and CYP 2C [12, 14], it is reasonable to assume that RP may induce P-gp or CYP 2C thus resulting in the decreased absorption of PHT. However, the unaffected ratio of the AUC of HPPH plus HPPH-G to that of PHT plus PHT-G (data not shown) can be inferred that the decreased absorption of PHT cannot be attributed to the enhanced metabolism mediated by CYP 2C. Subsequently, the possible involvement of P-gp in this interaction was investigated by using LS 180 cells to measure the effect of RP on the efflux transport of PHT. The result showed a decrease in intracellular accumulation of PHT in the presence of RP which indicated that the activity of P-gp could have been induced, which might explain the decreased absorption of PHT in rats. These results could also echo previous studies reporting that overexpression of P-gp could cause a decrease in PHT levels in the rats [18, 22].

While the rats were coadministered with RP in single dose or pretreated with seven doses, the significantly decreased *K*_10_ of PHT, PHT-G, HPPH, and HPPH-G indicated that eliminations of both the parent form and the metabolites were inhibited in rats. It has been reported that majority of the dose of PHT was excreted as HPPH-G and only small amount was excreted as PHT-G in human urine [23, 24], thus it could be assumed that renal excretion of HPPH-G and PHT-G might also involve renal MRP 2 like PHT [25, 26]. Therefore, it was suspected that RP might decrease the renal elimination of PHT, PHT-G, HPPH, and HPPH-G through the inhibition of MRP 2.

To explore the possible involvement of MRP 2 in this interaction, transport assays of PHT and HPPH in the presence and absence of Indo, an inhibitor of MRP 2, were conducted in MDCK II and MDCK II-MRP 2. The results showed that intracellular accumulation of PHT and HPPH was lower in MDCK II-MRP 2 than the MDCK II, thus suggesting that both PHT and HPPH were substrates of MRP 2. Moreover, the intracellular accumulations of PHT and HPPH in MDCK II-MRP 2 and MDCK II significantly increased by Indo had led to the confirmation that PHT and HPPH were substrates for MRP 2. To our knowledge, this is the first study reporting that HPPH is a substrate of MRP 2. Owing to the unavailability of PHT-G and HPPH-G, whether MRP 2 was involved in the efflux of PHT-G and HPPH-G could not be determined.

Our previous pharmacokinetic study of RP indicated that glucuronides/sulfates of aloe-emodin, rhein, emodin, and chrysophanol were the major molecules in the circulation, and rhein existed in part as free form [3, 8] which were putative substrates of MRPs. Therefore, the serum metabolites of RP (RPMs) were prepared and characterized for mimicking the molecules that interacted with MRP 2 in the kidney. A transport study was subsequently carried out using RPMs to measure the effect on the transport of PHT and HPPH in MDCK II-MRP 2. The increased accumulation of PHT and HPPH in MDCK II-MRP 2 indicated that the efflux activity of MRP 2 was inhibited by RPMs. It can be proposed that the G/S of various anthraquinones and rhein free form in RPMs, existing as anions under pH 7.4 and being putative substrates of MRP 2, are the causative agents that decreased the elimination rates of PHT, PHT-G, HPPH, and HPPH-G following coadministration of RP.

Although the elimination of PHT was inhibited by RPMs, the effect of decreasing the absorption of PHT caused by RP is much stronger than that on the elimination of PHT upon observing the serum profiles in Figure 2. Therefore, an overall effect of decreased systemic exposures to PHT, PHT-G, HPPH, and HPPH-G can be mainly attributable to a significant activation of P-gp by RP. Therefore, it is predicted that the combined therapy of RP with any western medicines which are P-gp substrates, such as digoxin and cyclosporine, can result in diminished efficacy. On the contrary, if RP is coadministered with any western medicines which are MRP 2 substrates rather than P-gp substrates, the efficacy or toxicity might be increased.

In conclusion, acute and chronic coadministration of RP can significantly decrease the systemic exposure of PHT, PHT-G, HPPH, and HPPH-G in rats mainly through activation of P-gp. Therefore, the results from this investigation conclude that caution will need to be exercised when RP and PHT are used concurrently.

## Figures and Tables

**Table 1:** Pharmacokinetic parameters of PHT, PHT-G, HPPH, and HPPH-G in six rats receiving oral PHT (200 mg/kg) alone and coadministration with single dose and seven doses of RP decoction (2 g/kg).

Parameters	Treatments		
	PHT alone	PHT + RP (2 g/kg)	PHT + RP (7th dose of 2 g/kg)
PHT			
*C*_max⁡_	15.7 ± 0.7^a^	7.5 ± 0.6^b^	7.3 ± 0.6^b^
		(−51.0 ± 5.1%)	(−53.1 ± 3.7%)
AUC_0–720_	4261.3 ± 146.8^a^	2650.0 ± 211.1^b^	2024.9 ± 209.0^b^
		(−36.9 ± 6.6%)	(−51.9 ± 5.3%)
*K*_10_	0.0039 ± 0.0002^a^	0.0028 ± 0.0003^b^	0.0025 ± 0.0002^b^
		(−27.9 ± 7.9%)	(−30.4 ± 6.2%)
PHT-G			
*C*_max⁡_	1.7 ± 0.2^a^	0.8 ± 0.1^b^	0.5 ± 0.1^b^
		(−51.7 ± 4.2%)	(−65.1 ± 10.4%)
AUC_0–720_	557.2 ± 59.9^a^	232.5 ± 37.9^b^	172.5 ± 22.1^b^
		(−51.7 ± 12.1%)	(−64.8 ± 8.5%)
*K*_10_	0.0033 ± 0.0003^a^	0.0019 ± 0.0002^b^	0.0023 ± 0.0003^b^
		(−51.5 ± 4.9%)	(−39.9 ± 7.3%)
HPPH			
*C*_max⁡_	4.5 ± 0.3^a^	2.5 ± 0.1^b^	2.3 ± 0.1^b^
		(−43.9 ± 4.6%)	(−46.3 ± 6.9%)
AUC_0–720_	1556.0 ± 81.5^a^	1058.3 ± 45.1^b^	954.0 ± 49.8^b^
		(−30.3 ± 5.7%)	(−37.5 ± 5.0%)
*K*_10_	0.0013 ± 0.0002^a^	0.0008 ± 0.0003^b^	0.0003 ± 0.0001^b^
		(−67.9 ± 13.8%)	(−76.6 ± 7.0%)
HPPH-G			
*C*_max⁡_	14.8 ± 0.7^a^	8.4 ± 0.5^b^	8.1 ± 0.5^b^
		(−42.5 ± 4.0%)	(−44.3 ± 4.2%)
AUC_0–720_	4482.9 ± 114.7^a^	3430.8 ± 208.5^b^	3099.9 ± 155.0^b^
		(−22.8 ± 6.3%)	(−30.6 ± 3.7%)
*K*_10_	0.0039 ± 0.0002^a^	0.0018 ± 0.0002^b^	0.0016 ± 0.0002^b^
		(−53.6 ± 3.9%)	(−59.3 ± 4.7%)

^a, b^ Significant difference at *P* < 0.05 denoted by different letters.

*C*_max⁡_ (*μ*g/mL): peak serum concentration.

AUC_0–720_ (*μ*g·min/mL): areas under the curves from time zero to the last point.

*K*_10_: the elimination rate (min^−1^).

Values are means ± SE.
